# Advanced biomedical hydrogels: molecular architecture and its impact on medical applications

**DOI:** 10.1093/rb/rbab060

**Published:** 2021-11-09

**Authors:** Jonathan T Peters, Marissa E Wechsler, Nicholas A Peppas

**Affiliations:** 1 McKetta Department of Chemical Engineering, The University of Texas at Austin, Austin, 200 E. Dean Keeton, Austin, TX 78712, USA; 2 Institute for Biomaterials, Drug Delivery, and Regenerative Medicine, The University of Texas at Austin, 107 W. Dean Keeton, Austin, TX 78712, USA; 3 Department of Biomedical Engineering and Chemical Engineering, The University of Texas at San Antonio, One UTSA Circle, San Antonio, TX, 78249, USA; 4 Department of Biomedical Engineering, The University of Texas at Austin, 107 W. Dean Keeton, Austin, TX 78712, USA; 5 Division of Molecular Pharmaceutics and Drug Delivery, College of Pharmacy, The University of Texas at Austin, 107 W. Dean Keeton, Austin, TX 78712, USA; 6 Department of Surgery and Perioperative Care, and Department of Pediatrics, Dell Medical School, The University of Texas at Austin, 1601 Trinity St., Bldg. B, Austin, TX 78712, USA

**Keywords:** hydrogels, networks, mesh size, hydrogel reactions, biological applications, biomedical applications

## Abstract

Hydrogels are cross-linked polymeric networks swollen in water, physiological aqueous solutions or biological fluids. They are synthesized by a wide range of polymerization methods that allow for the introduction of linear and branched units with specific molecular characteristics. In addition, they can be tuned to exhibit desirable chemical characteristics including hydrophilicity or hydrophobicity. The synthesized hydrogels can be anionic, cationic, or amphiphilic and can contain multifunctional cross-links, junctions or tie points. Beyond these characteristics, hydrogels exhibit compatibility with biological systems, and can be synthesized to render systems that swell or collapse in response to external stimuli. This versatility and compatibility have led to better understanding of how the hydrogel’s molecular architecture will affect their physicochemical, mechanical and biological properties. We present a critical summary of the main methods to synthesize hydrogels, which define their architecture, and advanced structural characteristics for macromolecular/biological applications.

## Monomers and hydrogel architecture

### Monomers

Synthetic hydrogels are highly reproducible with controlled chemical and physical properties that can be tailored for specific applications. The properties of hydrogels are dictated by the monomers used (Table 1) during synthesis [1]. The use of hydrophilic monomers allows for increased transmission of water and oxygen while maintaining the mechanical properties of the polymer backbone. Due to these properties, hydrophilic monomers are commonly used in drug-delivery devices and contact lenses.

**Table 1.** Hydrophilic monomers commonly used in synthesis of synthetic hydrogels for drug-delivery applications

Monomer chemical name	Monomer abbreviation	Properties
Hydroxyethyl methacrylate	HEMA	Hydrophilic, hydroxyl functional
Hydroxyethoxy ethyl methacrylate	HEEMA	Hydrophilic; hydroxyl functional
Hydroxydiethoxy ethyl methacrylate	HDEEMA	Hydrophilic; hydroxyl functional
Methoxyethyl methacrylate	MEMA	Low Tg monomer
Ethylene glycol dimethacrylate	EGDMA	Hydrophilic; hydroxyl functional
N-vinyl-2-pyrrolidone	NVP	Hydrophilic
N-isopropyl acrylamide	NIPAAm	Thermosensitive
N'N'-diethyl acrylamide	DEAAm	Thermosensitive
Vinyl acetate	VAc	Hydrophilic
Acrylic acid	AA	Hydrophilic; acid-containing
Methacrylic acid	MAA	Hydrophilic; acid-containing
N-(2-hydroxypropyl) methacrylamide	HPMA	Hydrophilic; non-immunogenic
Ethylene glycol	EG	Hydrophilic; H-bonding site
PEG acrylate	PEGA	Monofunctional; used for PEG grafts
PEG methacrylate	PEGMA	Monofunctional: used for PEG grafts
PEG diacrylate	PEGDA	Hydrophilic cross-linking agent
PEG dimethacrylate	PEGDMA	Hydrophilic cross-linking agent

For biomedical applications, poly(ethylene glycol) (PEG) is often utilized due to its unique characteristics—non-toxic, non-immunogenic and stealth properties. Ethylene glycol-containing monomers are highly hydrophilic with multiple hydrogen-bonding sites. The incorporation of PEG into polymeric drug systems can also be used to modulate sustained release of drugs. Additionally, PEGylation of drugs and therapeutic proteins can increase their circulation time, which improves their pharmacokinetics and pharmacodynamics. Hydroxyl-containing monomers can also serve as hydrogen-bonding sites, as well as provide polymers with compatibility for water and polar solvents.

Acid-containing hydrophilic monomers, particularly acrylic acid (AA) and methacrylic acid (MAA), are typically used to improve the solubility of polymers in aqueous media. In addition, acid functional groups can promote adhesion through hydrogen bonding. Such monomers are often used to modify hydrogel properties, as in the case of pH-responsive hydrogel formulations [2]. These hydrogels, which contain both hydrogen-bonding donors and acceptors, can form reversible interpolymer complexes due to hydrogen bonding. Drug-delivery systems can take advantage of the formation/dissociation of such complexes, which affect hydrogel swelling. A notable example of such is poly(methacrylic acid) hydrogels grafted with PEG, which have been engineered for intestinal drug delivery due to its pH-responsive swelling behavior [1, 3–5]. In this specific example, the hydrogel remains collapsed in the acidic conditions of the stomach (protecting the biological payload), and swells in the conditions of the small intestine promoting absorption of the delivered drug.

### Cross-linking agents

The integrity of hydrogels is maintained by physical and/or chemical cross-links between polymer chains [6–8]. Chemical cross-linking strategies form covalent bonds between polymer chains. As a result, chemically cross-linked hydrogels have good mechanical stability and degrade by breaking labile bonds. An important aspect to note is many cross-linking agents are toxic compounds that must be removed from hydrogels before use in biomedical applications.

Physically cross-linked gels exploit non-covalent interactions, such as hydrophobic interactions, hydrogen bonding, chain entanglement, crystallinity and ionic complexation. While physical cross-linking methods can be advantageous for simple synthesis without requiring chemical modification or toxic agents, applications *in vivo* can be limited due to poor mechanical stability. However, there are many other physically cross-linked hydrogels, which have demonstrated outstanding physical properties for biomaterial applications [9–11]. Additionally, measurement of mesh size, degradation or chemical functionalization can be more difficult to determine, limiting the design flexibility of physical gels for pharmaceutical applications [6].

Radical polymerization is a common strategy to obtain chemically cross-linked gels from low molecular weight monomers with polymerizable functional groups, such as acrylate or methacrylate moieties. Incorporation of an initiator that decomposes in response to a stimulus, such as exposure to UV or visible light (photopolymerization) [12] or oxidation–reduction (e.g. the ammonium persulfate/N, N, N′,N′-tetramethyl ethylenediamine initiator pairing) [13] generates free radicals that enable formation of a cross-linked network. Another strategy to form cross-links is to take advantage of functional groups on water-soluble monomers or polymers (e.g. hydroxyl groups, carboxylic acids and amines) via chemical reaction of complementary groups. For example, glutaraldehyde is a common agent used to form cross-links with monomers or polymers containing hydroxyl groups, such as poly (vinyl alcohol) [14].

Condensation reactions between hydroxyl groups or amines with carboxylic acids are also frequently used [15]. Michael addition type reactions are another emerging method for chemical cross-linking suitable for gel formation at room temperature and physiological pH, particularly for injectable materials [16]. ‘Click’ chemistry between azides and acetylenes is a desirable method due to the high specificity and more controlled distribution over cross-linking, and therefore mesh size, than other previously mentioned methods. Traditional copper-catalyzed click chemistry is limited for biomedical application by the intrinsic toxicity of the synthesis, however, progress has been made toward the development of copper-free reactions that occur more slowly but are acceptable for potential patient administration [17, 18].

For many applications it is advantageous for the hydrogel to degrade, which can be achieved by inclusion of the appropriate starting materials and cross-linking strategy. Degradable hydrogels allow for modulation of specific drug release profiles, prevent follow-up procedures for retrieval of materials, and can further enable targeting strategies to specific areas in the body [19, 20]. The two most common strategies for cleavage of polymer chains are by either hydrolytic or enzymatic degradation. For example, the colon is rich in reductive (e.g. azo-reductase) and hydrolytic (e.g. glycosidase) enzymes, including the enzyme dextranase. Hovgaard *et al.* [21] first exploited the physiological environment by designing dextran hydrogels for oral delivery of the anti-inflammatory agent hydrocortisone. An alternative approach, also for colonic-delivery, utilized an azoaromatic cross-linking agent in pH-responsive gels for delivery of insulin [22]. More recently, specific peptide sequences have been incorporated as cross-linking agents into hydrogels to achieve selective delivery, including cellular responsive gels rendering hydrogel delivery systems degradable at sites of inflammation [23], or other disease-specific enzymes [24].

### Cross-linked structure and mesh size calculations

The mesh size, *ξ*, is defined as the linear distance between two adjacent cross-links, as calculated by two methods. The derivation of the mesh size calculation starts with Equation 1.1.

1.1 ξ=αro¯212.

Where *α* is the extension or elongation ratio of the polymer chains and ro¯212 is the root-mean-square, unperturbed, end-to-end distance of the polymer chains between two neighboring cross-links. For isotropically swollen hydrogels, the extension ratio can be determined from the swollen polymer volume fraction, $v_{2,s}$, as shown in Equation 1.2.

1.2 α=υ2,s-13.

The swollen polymer volume fraction is determined experimentally as the solvent parameters affect the swelling behavior of the hydrogel. The unperturbed end-to-end distance can be calculated using Equation 1.3.

1.3 ro¯212=l 2CnMc¯Mr1/2.

Where l is the bond length along the polymer backbone (1.54 Å for vinyl polymers), $C_{n}$ is the Flory characteristic ratio (tabulated for many polymers), $\bar{M_{C}}$ is the average molecular weight between cross-links and $M_{r}$ is the molecular weight of the repeat units of the polymer chain. By combining Equations 1.1, 1.2 and 1.3, the mesh size of a swollen hydrogel is a more frequently determined using Equation 1.4.

1.4 $$\xi ={\upsilon_{2,s}^{-1/3}\left(\frac{2C_{N}\bar{M_{C}}}{M_{r}}\right)}^{1/2}l.\;$$

Methods to experimentally determine $\bar{M_{C}}$ include the use of the Peppas–Merrill, the Brannon-Peppas or modified Brannon-Peppas equations according to the number of ionizable groups.

## Physical, chemical and toxicological properties of hydrogels

### Factors affecting hydrogel swelling

The degree to which a hydrogel swells is dependent on a variety of factors. One of the eminent influences is the fundamental chemical nature of the polymer. Monomers that impart high levels of hydrophilicity will yield substantially higher swelling ratios in water as compared to more hydrophobic constituents. Hydrophilicity typically results from favorable van der Waals interactions with the dipolar water molecules that comprise the surrounding medium. Thus, the hydrophilicity of a hydrogel, and therefore, the swelling ratio, can be increased by ionization of constituent groups in polyelectrolyte gels. As a result, polyelectrolyte gels frequently demonstrate significantly higher degrees of swelling than non-electrolyte gels, often imbibing up to several hundred times their dry polymer weight at equilibrium [25–29].

Systems based on various polyacrylates or polyacrylamides offer such electrolytic behavior and are frequently used as ‘superabsorbent polymers’, currently considered the most commercially successful family of hydrogels [27], where their high water-uptake capability is widely utilized in diapers and other personal care products that absorb high quantities of various bodily fluids. Cipriano *et al.* [30] utilized N,N-dimethylacrylamide (DMAA) copolymerized with sodium acrylate to achieve swelling ratios of up to 3000, while simultaneously being able to tolerate strains of up to 400% before rupture. No added multifunctional cross-linkers were used, relying instead on the self-cross-linking ability of DMAA to create a uniform distribution of cross-link points, which in turn leads to the improved mechanical properties of the gels at very high swelling ratios. Because of the combination of extremely high water uptake and mechanical integrity, such hydrogels may open up novel applications for hydrogels in medicine and industry, as materials generally lack one of either ‘soft’ biomaterial properties or high tensile strength.

Beyond the fundamental chemistry of the monomers present in the hydrogels, hydrogel swelling is also affected by the cross-linking density of the hydrogel. As the cross-linking density increases (i.e. more cross-linking tie points per volume of hydrogel), the free polymer chains become more entangled with one another and have less translational freedom, decreasing the entropic change of mixing. As a result, hydrogels swell to a lesser extent when highly cross-linked. Modeling of this phenomenon was first performed by Flory [31–33] and later modified by Peppas and Merrill [34] and Brannon-Peppas [35] to describe hydrogel swelling as a function of $\bar{M_{c}}$, the average molecular weight between cross-links.

As cross-linking density increases, $\overset{-}{M_{c}}$ decreases, and the two are inversely related shown in Equation 2.1.

2.1 $$X=\frac{\bar{M_{r}}}{2\overset{-}{M_{c}}}.$$

**Figure 1.**
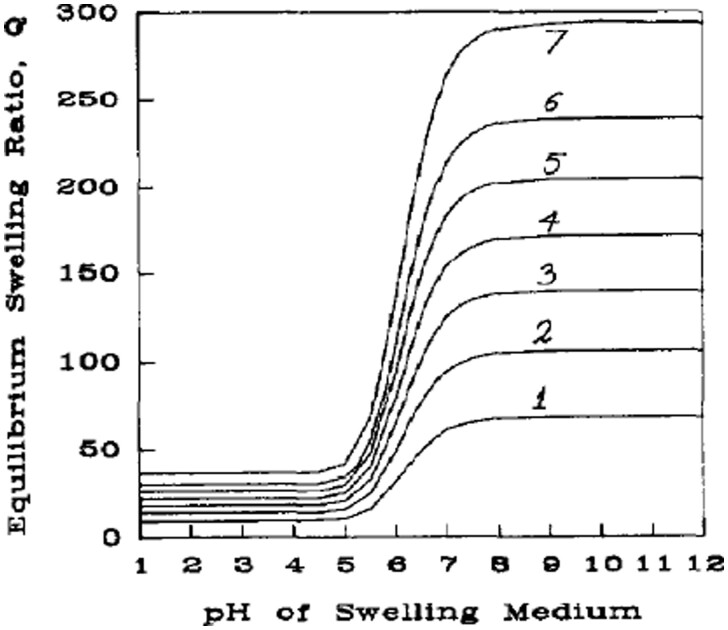
Theoretical swelling predictions at comparable ionic strength conditions for an anionic network with: (1) $\overset{-}{M_{C}}$ = 2000, (2) $\overset{-}{M_{C}}$ = 4000, (3) $\overset{-}{M_{C}}$ = 6000, (4) $\overset{-}{M_{C}}$ = 8000, (5) $\overset{-}{M_{C}}$ = 10 000, (6) $\overset{-}{M_{C}}$ = 12 000 and (7) $\overset{-}{M_{C}}$ = 15 000. Reproduced with permission from Ref. [35]

Where *X* is the cross-linking density and $\overset{-}{M_{r}}$ is the average molecular weight of the repeat unit. Therefore, with increased cross-link density, $\overset{-}{M_{c}}$ will be reduced, and based on the Brannon-Peppas equation for polyelectrolyte hydrogels, solution of the swelling ratio in terms of $\overset{-}{M_{c}}$ (inversely proportional to *X*) shows a decrease in volumetric swelling ratio (*Q*) with increasing cross-linking density [36], as shown in Fig. 1 [35]. As a result of this strong relationship, cross-linking density has been widely used as a tunable property for tailoring hydrogels to specific porosities for applications in drug delivery [37–44].

**Figure 2.**
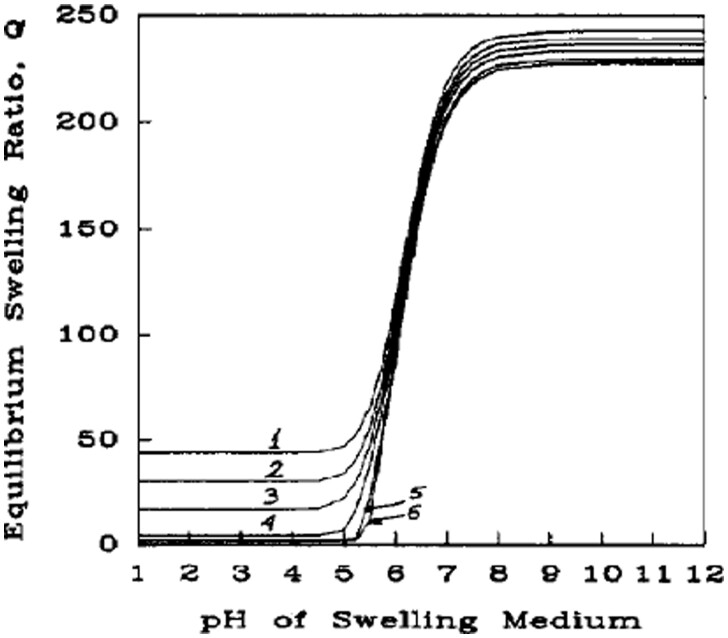
Theoretical swelling predictions at comparable ionic strength conditions for an anionic network with: (1) χ = 0.1, (2) χ = 0.3, (3) χ = 0.45, (4) χ = 0.6, (5) χ = 0.8 and (6) χ = 0.9. Reproduced with permission from Ref. [35]

Finally, hydrogel swelling is affected strongly by the surrounding medium. To some extent, this is merely the inverse of saying that hydrogel swelling is affected by the chemistry of the hydrogel, since affinity for the medium is the determining factor. This aspect of the swelling is often summed up through the Flory solvent interaction parameter, χ, which describes the attractive or repulsive interactions between the solvent and the polymer. As seen in Fig. 2, there is a dependence of swelling ratio on χ, although it is not as substantial as factors such as cross-linking density [35].

**Figure 3.**
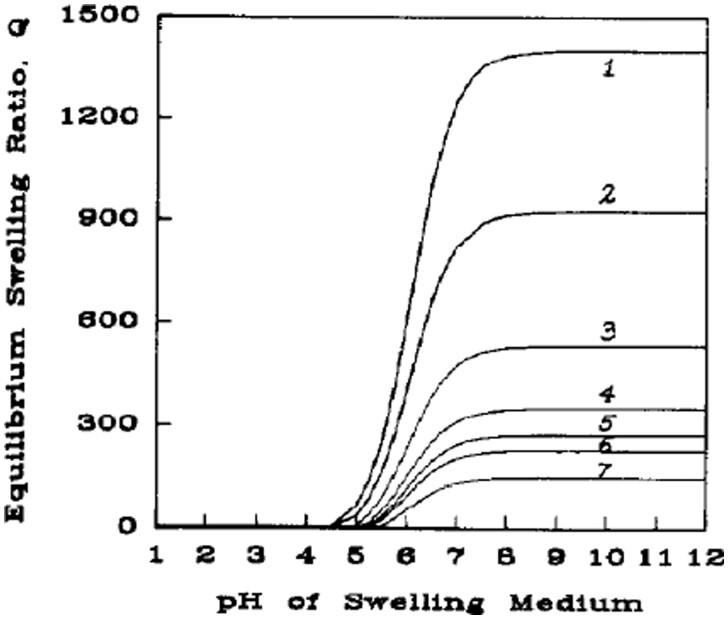
Theoretical swelling predictions at ionic strength (*I*) conditions for an anionic network with: (1) *I* =0.05, (2) *I* =0.1, (3) *I* =0.25, (4) *I* =0.5, (5) *I* =0.75, (6) *I* =1.0 and (7) *I* =2.0. Reproduced with permission from Ref. [35]

**Figure 4.**
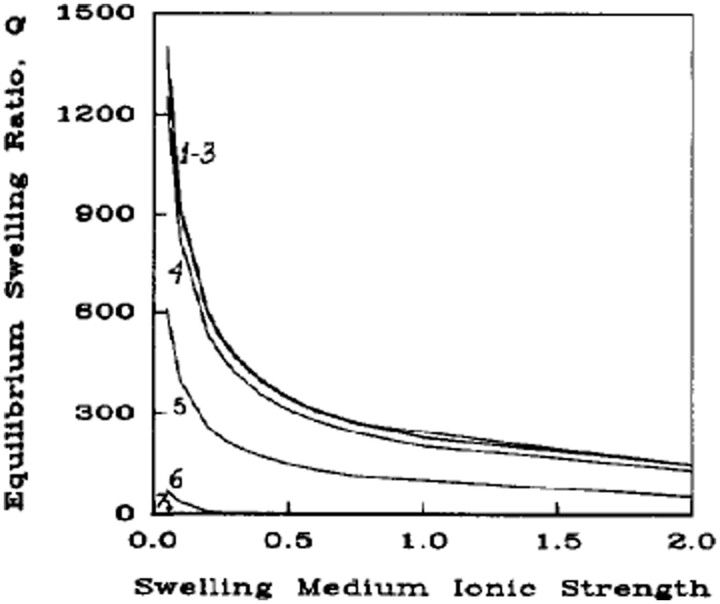
Theoretical swelling predictions at comparable ionic strength conditions for an anionic network with: (1) p*K*a=2.0, (2) p*K*a=4.0, (3) p*K*a=5.0, (4) p*K*a=6.0, (5) p*K*a=7.0, (6) p*K*a=8.0 and (7) p*K*a=10.0. Reproduced with permission from Ref. [35]

Another aspect of the surrounding medium, which has a far more substantive effect on the equilibrium swelling of the hydrogel is the ionic strength of the solvent. The ionic strength of the medium strongly affects the chemical potential of the system in polyelectrolyte hydrogels through ionic interactions that can generate strong osmotic forces that require medium imbibition or expulsion [36]. This contribution to hydrogel swelling has been modeled by Brannon-Peppas and Peppas [35], and theoretical solutions of this model for swelling ratio as a function of ionic strength (as seen in Figs 3 and 4) show a strong, non-linear relationship of decreased ionic strength leading to increased swelling. This same phenomenon has been shown in actual systems and exploited for enhancing efficacy in various applications [45–56].

### Dynamics of swelling behavior

The dynamics of swelling and diffusional solute release from hydrogels have been well-studied and modeled [57–61]. The dynamics are generally classified and modeled based on the relative rates of solvent diffusion through the hydrogel matrix and polymer chain relaxation. When the rate of solvent diffusion into the hydrogel is significantly slower than the rate of hydrogel relaxation, the rate of swelling is diffusion-limited, and the hydrogel exhibits Fickian swelling behavior. On the other hand, if the rate of polymer chain relaxation is significantly slower than solvent diffusion through the matrix, the rate of swelling is relaxation-limited, and the hydrogel exhibits non-Fickian swelling behavior.

While a rigorous modeling of the swelling behavior requires solution of full, 3D transport equations with appropriate initial and boundary conditions, useful approximations of the swelling behavior provide more tractable methods for analyzing the swelling response [36]. These approximations are based on the use of a dimensionality index, *d*, and empirical Peppas parameters, *k* and *n* [58, 60, 61]. The dimensionality index, *d*, is a measure of the number of directions in which hydrogel expansion may occur. For a system exhibiting isotropic swelling in three dimensions, the value of *d* is three. However, hydrogel swelling may be restricted by imposed boundary conditions through mechanical means or coatings that prevent swelling in one or more dimensions. For example, in the case where a hydrogel disk is covered on one of its surfaces, the dimensionality index is reduced to two. For systems exhibiting anisotropic swelling, non-integer values of *d* may result, most often ranging between 2.5 and 3 for hydrophilic systems, or between 2 and 2.5 for less hydrophilic systems [36, 58].

With the dimensionality index, one can relate the volume (*V*) and surface area (*A*) changes of a hydrogel exhibiting Fickian swelling behavior to the unrestricted volumetric swelling ratio, *Q*, as follows.

2.2 $$\frac{V_{\mathrm{gel}}}{V_{\mathrm{dry}}}=Q^{d/3}$$

2.3 $$\frac{V_{\mathrm{gel}}}{V_{\mathrm{dry}}}=Q^{d/3}$$

2.4 $$\frac{A_{\mathrm{gel}}}{A_{\mathrm{dry}}}=Q^{\left(d-1\right)/3}.$$

Combining these relations with the time-dependent relations proposed by Ritger and Peppas [61], the volume and surface area of the gel at a given time may be modeled as:

2.5 $$\frac{Q(t)}{Q_{\infty }}=kt^{n}$$

2.6 $$\frac{V_{\mathrm{gel}}(t)}{V_{\mathrm{dry}}}=Q_{\infty }^{d/3}\;k^{d/3}\;t^{nd/3}$$

2.7 $$\frac{A_{\mathrm{gel}}(t)}{A_{\mathrm{dry}}}=Q_{\infty }^{(d-1)/3}\;k^{(d-1)/3}\;t^{n(d-1)/3},$$

where *Q_∞_* is the equilibrium volumetric swelling ratio at the end conditions. The empirical values of *k*, *d* and *n* can be determined from these relations and standard regression techniques [36]. The exponent *n* is termed the diffusional release exponent, and its value is an indication of the relative rates of diffusion and polymer relaxation. For pure Fickian water diffusion, *n* is expected to be 0.43 [61], although any value near 0.5 is considered to be Fickian-controlled. Values above 0.5 are indicative of non-Fickian, relaxation-controlled swelling or drug release [60, 61]. These same equations are often used to model drug release from hydrogel carriers as well, substituting the swelling ratios for the amount of drug released, although the constants will have different values due to the diffusive species being a drug molecule rather than the medium alone.

### Mechanical properties

The mechanical behavior of a hydrogel plays an important role in its functionality and applications. The mechanical properties of a hydrogel greatly depend on the monomer composition (e.g. rigidity of polymer chains), the cross-linking type and density and the polymerization conditions [62]. For hydrogels in drug-delivery systems, mechanical properties affect drug release, as well as the product’s shelf life. Mechanical strength, along with degradability, diffusivity and other physical properties, depend on the mesh size of the hydrogel network. The structure and the mesh size of swollen hydrogels can be tailored for desired release profiles of a variety of molecules [36, 63].

The rubber elastic behavior of hydrogels can be determined using tensile testing. Most swollen hydrogels exhibit elastic behavior, such as high extensibility and recovery after deformation. However, hydrogels tend to perform as viscoelastic materials, meaning that the movement in the polymer chains due to applied mechanical stress results in a time-dependent recovery after deformation is removed. The time-dependent viscoelastic behavior can be determined by dynamic mechanical thermal analysis. The elastic or storage modulus and loss modulus can be measured by the mechanical response of a sample under periodic stress or strain. In determining the mechanical properties of hydrogels, it is important to control temperature by using an environmental chamber and to prevent water loss by using a petroleum gel or silicone coating. Hydrogels can be tested using *in situ*, or their mechanical properties can be extrapolated at such conditions to provide an understanding of how a hydrogel-based device will function for specific applications [62].

### Hydrogel structure and its influence in biomedical applications

#### Cytotoxicity and in vivo toxicity

Despite the fact that a lot of interest and effort is being placed into the development of hydrogel-based biomedical applications, the level of translational clinical output is still limited by the uncertainty of their toxicological profiles. It is well established that the unreacted monomers, oligomers and initiators that might contaminate and leach out of the hydrogel during usage are the main source of toxicity associated with these carriers. Structure–activity relationships of the mechanisms of methacrylate-induced toxicity have been known for some time [64–67] and were revisited with using advanced testing strategies, such as quantum chemical descriptors and computational chemistry [68]. It is imperative to rule out any adverse effects that hydrogels might generate before they can be safely translated to the clinic.

However, due to the lack of a general consensus on critical characterization parameters, shortage of harmonized protocols to support testing, and the vast variety of engineered materials, their translation into clinic is particularly complex. The US Food and Drug Administration (FDA) has been launching several International Council for Harmonization of Technical Requirements for Pharmaceuticals for Human Use guidelines on the safety of pharmaceutics and medical devices [69]. The European Commission has bundled its guidelines in the Registration, Evaluation and Administration of Chemicals regulation. This regulation was plainly adopted for nanotoxicity evaluation even though it was initially designed for chemical substances [70].

The major issues with current *in vitro* cytotoxicity methods are: (i) the lack of consensus on the dose metric, (ii) the lack of standardization and guidelines on how to perform an *in vitro* toxicological evaluation, (iii) the possibility of hydrogels interfering with assays and (iv) the shortcomings inherent to the most used classical 2D monocultures. Features, such as high adsorption capacity, hydrophobicity, surface charge, optical and magnetic properties, or catalytic activity may interfere with assay components or detection systems. For instance, the MTT and lactate dehydrogenase colorimetric assays present several limitations due to the pH-dependence of the substrate, interference of metal ions and of materials with optical properties [71, 72].

New *in vitro* approaches for toxicity evaluation of new biomedical materials, especially nano-based materials, are receiving a lot of attention. For instance, high throughput screening and high content screening approaches for the evaluation of multiple endpoints via multiple assays preferably in multiple cell types from different organisms are under focus [73]. In addition, new model systems that minimize particle sedimentation (inverted cell models, flow and microfluidic systems) and mimic intercellular communication (cellular co-cultures, 3D models) are also under current investigation as an attempt to develop more reliable *in vitro* models with higher predictive power, mimicking the *in vivo* environment more closely [71].

Subsequently, *in vivo* studies are most commonly performed on rodents due to low cost and easy manipulation. Acute, sub chronic and chronic toxicity, genotoxicity, carcinogenicity, immunotoxicity, hemotoxicity, skin and eye irritation or corrosion and toxicokinetics are the aspects that should be considered [74, 75]. One of the most used techniques for *in vivo* evaluation of toxicity is the histopathological examination of selected organs and tissues from a sacrificed animal. Acute oral toxicity, defined as the adverse effects that occur within a relatively short time after oral administration of a single dose or multiple doses of a substance in 24 h, can be estimated by using the maximum tolerated dose method. In this method, hydrogels are orally administered to animals, which are then continuously observed for 14 days for general conditions (hair, feces, energy, activity, behavior pattern and other clinical signs), after which blood and serum are collected for routine hematology and biochemistry analysis [76]. The hemolytic activity of hydrogels has been tested by determining the hemolysis caused by hydrogels in contact with human blood [77].

In order to reduce the number of animals used for *in vivo* toxicity testing but still provide valuable information that can fill the *in vitro*-*in vivo* gap, more reliable *in vitro* models with higher predictive power are needed. Understanding the immune compatibility of hydrogel formulations is also one of the important factors in (pre)clinical development and requires reliable *in vitro* and *in vivo* immunotoxicity tests [78]. The generally low sensitivity of standard *in vivo* toxicity tests to immunotoxicities, inter-species variability in the structure and function of the immune system, high costs and relatively low throughput of *in vivo* tests and ethical concerns about animal use underscore the need for trustworthy new assays.

## Stimuli-responsive biomedical systems

### pH-responsive systems

Hydrogels that respond to perturbations in the pH of their external environment are designated as pH-responsive systems and are a subset of the broader class of stimuli-responsive hydrogels [79, 80]. This response can range from pH-induced swelling/deswelling to the pH-dependent degradation of the polymer network. Systems that can respond to variations in surrounding pH are of particular interest for pharmaceutical applications due to the natural variations of pH within the body, such as within the gastrointestinal tract (GI) [81, 82], extracellular compartments [83, 84], and vagina [85] as well as variations of pH observed in disease states like cancer [86–88] and inflamed tissue [89].

The pH-dependent swelling response of hydrogels is due to the ionization or deionization of pendant ionic groups located on the polymer backbone [79]. The electrostatic repulsive forces generated by the charged polymer backbone initiate the pH-dependent swelling/deswelling driven by the diffusion of water into or out of the network [3, 79, 90]. Anionic hydrogels contain ionic pendant groups that are ionized at a pH greater than their acid dissociation constant, p*K*a. Therefore, they are charged and, henceforth, swell at pH > p*K*a. Commonly used monomers to incorporate an anionic swelling behavior into the hydrogel network include AA and MAA. Both monomers have pendant carboxylic acid groups that retain their hydrogen ions at pH < p*K*a but lose it once the pH increases above the p*K*a resulting in a negatively charged polymer backbone, which leads to electrostatic repulsion, water imbibition and subsequent swelling. Conversely, cationic hydrogels have ionic pendant groups that are ionized at a pH values less than their p*K*a, thereby swelling when pH < p*K*a. Common cationic monomers include dimethylaminoethyl methacrylate, diethylaminoethyl methacrylate and acrylamide (AAm) [91, 92].

In addition to the synthetic monomers listed, there are a number of naturally derived polymers, which also demonstrate pH-dependent responses, such as, albumin [93], gelatin [94], alginate [95, 96] and chitosan [97]. These pH-dependencies can be harnessed to form natural pH-responsive polymer networks that are dictated by the relationship between the pH of the surrounding medium and the isoelectric point (pI) of protein-based hydrogels [79] or the hydrophobic and charge interactions of polysaccharide-based systems [98]. Naturally derived hydrogel networks, as compared to their synthetic counterparts, can have reduced immunogenicity, as well as an inherent biodegradability, that is useful for the development of drug-delivery vehicles and drug-eluting implanted biomaterials [99].

Ionic networks have shown vast utility for pharmaceutical applications ranging from controlled-release drug-delivery systems for proteins, nucleic acids and small molecules, to prodrugs and adjuvants for vaccines [3, 4, 99–109]. The ability to incorporate pH-responsive moieties with a large variety of both natural and synthetic monomers into a responsive network allows researchers to tightly tune and refine the pH-response, and other hydrogel properties, to the specific application of interest, providing both control and intelligence to the designed systems [97, 99, 101, 102, 110–112].

### Temperature-responsive systems

Temperature responsive hydrogels have been investigated as drug-delivery vehicles due to the relatively constant physiological temperature environment [113–116]. These materials are classified into two categories based on their swelling response. Hydrogels, which exhibit an upper critical solution temperature (UCST), swell in response to an increase in temperature, in contrast, hydrogels that possess a lower critical solution temperature (LCST) collapse as temperature increases.

Most UCST-based hydrogels are developed around the interpenetrating network (IPN) system developed by Okano. This system involves an IPN of a carboxylic acid-containing monomer, such as MAA [13]. There have several other polymer formulations that have been developed which exhibit a UCST; however, few of these systems have been used to synthesize hydrogels [117]. However, if the ionic strength and pH of the surrounding hydrogel media are not controlled, the temperature responsive swelling behaviors can be adversely affected [118]. The great variation of these characteristics *in vivo* limits their potential application.

**Figure 5.**
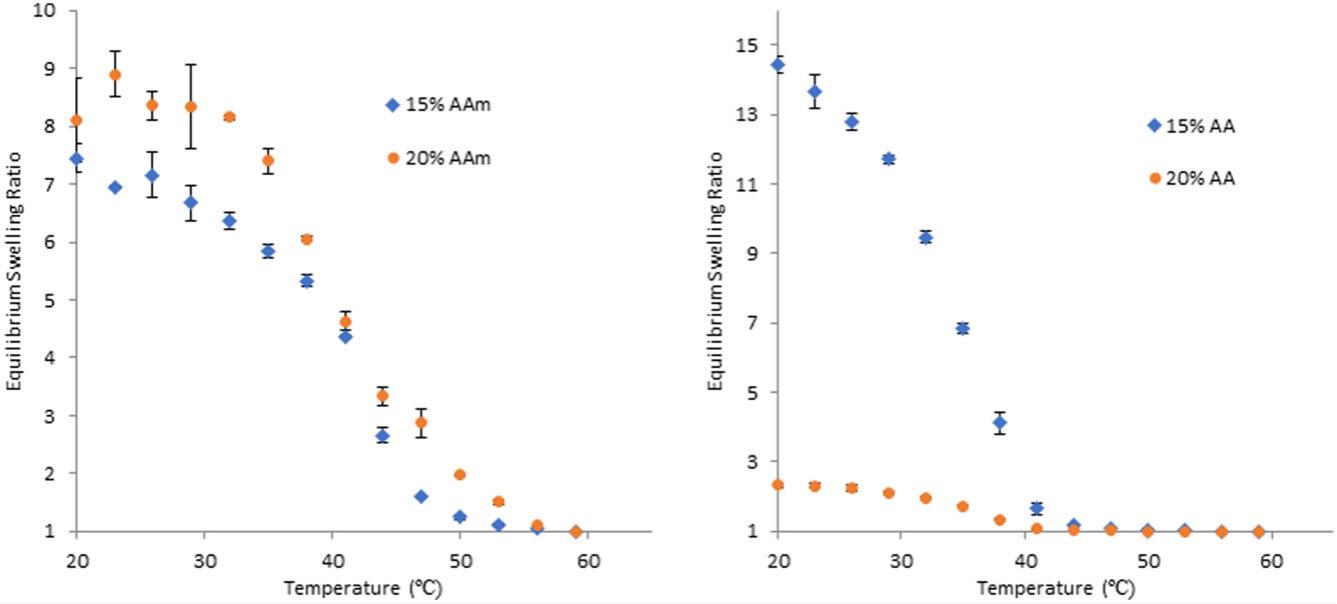
Equilibrium swelling ratio with increasing temperature of P(NIPAAm-co-Acrylamide)(left) P(NIPAAm-co-Acrylic acid)(right). Percentages are molar percentages of total monomer concentration in mol%. Equilibrium swelling ratio = (*d*/*d*_60_)^3^. Acrylamide (AAm), acrylic acid (AA)

LCST-based polymers have received increased attention due to more robust synthesis methods and a lesser dependence on solution characteristics compared to UCST-based polymers. While many LCST systems are based on poly(N-isopropylacrylamide) (PNIPAAm), copolymers are often added to modify the LCST and imbue polymers with added characteristics (Fig. 5) [83, 119, 120]. However, the addition of hydrophobic or hydrophilic copolymers can decrease or increase the LCST, respectively [121]. The addition of a co-monomer with other stimuli-responsive characteristics, such as photo or pH, can yield dual responsive materials. However, if these comonomers are included at too high of ratios, it can eliminate the responsiveness of the material entirely [121]. Another solution is to tune the LCST by copolymerizing with other LCST polymers, such as N,N-diethyl acrylamide and N-isopropyl methacrylamide, which have LCSTs above the normal physiological temperature [122]. More recent thermogelling polymers have been synthesized to create block copolymers, are injectable, degradable, form *in situ*, and can be used for a range of biomedical applications [123–126].

### Analyte-responsive systems

Numerous systems have been engineered to elicit stimuli-sensitivities to a particular analyte. Through a suitable transduction pathway, hydrogels may be synthesized to demonstrate responsive swelling behaviors upon recognition of almost any target molecule using one of the previously described modes of inducing molecular rearrangement. One interesting, and useful, class of analyte-responsive hydrogels is glucose-responsive hydrogels. Inducing a swelling or degrading response upon exposure to glucose enables potential hydrogel use for long-term insulin drug depots that release appropriate amounts of insulin in response to elevated blood sugar levels. This behavior would thus, mimic the body’s natural insulin release preventing the need for frequent blood pressure monitoring and injection for patients with diabetes [127].

Three independent mechanisms for glucose-sensitive hydrogel systems have been developed: glucose-oxidase (GOx)-based gels, concanavalin A (Con A)-based gels and phenylboronic acid (PBA)-based gels [128]. GOx-based gels rely on immobilization of glucose oxidase within pH-sensitive hydrogel matrices; when blood glucose levels get sufficiently high, glucose will diffuse into the hydrogel matrix, where GOx will catalyze the following reaction of glucose with oxygen, producing gluconic acid, shown in Equation 3.1 [129].

3.1 $$\mathrm{Glucose}+O_{2}+H_{2}O\overset{\mathrm{GOx}}{\to }\mathrm{Gluconic}\;\mathrm{Acid}+H_{2}O_{2}.$$

The resulting gluconic acid reduces the pH inside the hydrogel, causing the hydrogel to swell or deswell, eliciting insulin release by diffusion or convection, respectively. This strategy has been widely studied and improved upon to create hydrogels that are highly sensitive to glucose [129–140].

Con A-based gels rely on the immobilization of Con A, a lectin that binds various carbohydrates, throughout a hydrogel matrix that contains glycosylated pendant groups. In the hydrogel prior to glucose exposure, Con A binds to the pendant groups, causing additional cross-linking points that yield small pore sizes and entrap insulin within the matrix. When glucose enters the hydrogel, it competitively displaces the glycosylated pendants from Con A, reducing the number of effective tie points and therefore, resulting in insulin release by either swelling or a gel–sol transition, depending on whether the gel was chemically cross-linked or physically cross-linked with Con A, respectively [141–148].

Finally, PBA-based gels form reversible covalent complexes with diols present in saccharides like glucose [149–151]. Formation of these complexes results in a cationic charge on boron, which shifts the equilibrium of charges in the polymer backbone to become positive. The resulting charge yields increased hydrophilicity and ionic repulsion within the hydrogel, which leads to a swelling response that releases entrapped insulin. PBA gels do not require immobilized enzymes, so they are considered the most promising of these three classes of systems because they are not prone to degradation or diffusive loss of the responsive component from the hydrogel that would lead to loss of functionality. As such, many systems have been developed as drug-delivery depots or glucose sensors [152–160].

Numerous other analytes have also been used as targets to elicit stimuli-sensitive responses. Enzymes present in the body are suitable analytes, which can induce a hydrogel’s response in select environments for targeted drug-delivery applications. Typically, this is accomplished using peptide sequences that are degraded by the target enzyme [161, 162]. Kopecek and colleagues [163–172] have developed and tested N-(2-hydroxypropyl)methacrylamide hydrogels containing bound drug molecules attached via oligopeptide cross-links. The oligopeptide sequence is targeted by specific enzymes. For example, the oligopeptide sequence GFLG enables specific cleavage by cathepsin B, which is found in lysosomes. These systems utilize the peptide as a link between the drug and the polymer backbone, while other systems utilize the peptide as the hydrogel crosslinker, enabling targeted degradation of the hydrogel upon enzymatic reactions. The peptide sequence QPQGLAK has been used by Kim and Healy [173] to develop matrix metalloproteinase (MMP) degradable extracellular matrix scaffolds that degrade upon production of MMP-13 when osteoblasts begin forming new bone tissue. This enables degradation of the extracellular matrix (ECM) only after the osteoblasts produce the biological feedback signal, keeping the ECM around for sufficient time to induce healing, but degrading after healing begins as not to interfere with the full bone healing process. West and Hubbell similarly demonstrated the use of peptide-PEG-peptide block copolymers as an artificial ECM where the peptide sequence APGL was sensitive to collagenase, and the sequence VRN was sensitive to plasmin [174]. All of these systems, and many others, incorporate the peptide by acrylation with acryloyl chloride, enabling polymerization as a monomer unit in a standard chain polymerization reaction, although any other suitable cross-linking chemistry would also suffice [175, 176].

As the above examples show, nearly any analyte can be used to induce a hydrogel response with a suitable detection and transduction method. Molecularly imprinted polymers (MIPs) are rapidly expanding as a potential method for achieving specific analyte recognition in hydrogels [177–187]. MIPs are typically formed by mixing the target analyte with monomers chosen to display physical interactions with the target, allowing them to bind to recognition sites, and polymerize the gel around the analyte with high cross-linking densities [188–190]. This procedure forms a hydrogel around the analyte, while the high cross-linking density maintains the network structure from experiencing significant loss due to entropic mixing and thus, provides binding sites for the analyte at later times.

MIPs provide the sensing capability needed for analyte responsiveness, but a method for transduction is required prior to use [191–193]. With ingenuity, the binding could be made to elicit a swelling response, as with the PBA hydrogels for glucose detection described earlier. However, previous studies have used external sensing technologies, such as electrical current, voltage, changes in capacitance [194–197], infrared spectroscopy, Raman spectroscopy [198], quartz crystal microbalance and changes in refractive index [199–202]. Using these methods, binding of the analyte is sufficient to induce detectable changes using external sensors. With the MIP technology, researchers have demonstrated analyte-specific responses for many targets including, trypsin [203], cholesterol [204], theophylline [205], diazepam [205], morphine [206], corticosteroid [207], S-propranolol [208, 209], uric acid [210, 211] and many others.

With the advancement of MIP and continued ingenuity in materials chemistry, the range of analyte-responsive materials will doubtlessly continue to expand. The ramifications for therapeutic applications are broad and apparent, as *in vivo* detection of disease-specific markers could be immediately met with proper treatment options if such screening and diagnostic approaches were readily available. Analyte-responsive hydrogels will have significant impact in healthcare as inexpensive and stable biosensors [193, 212].

### Photoresponsive systems

Photochemical reactions show great promise for use in hydrogels. The high level of control that lasers can exhibit, and new two photon patterning, has led to great leaps in 3D patterning resulting in improved user defined control [213]. The one major drawback to photoresponsive hydrogels is their limited effectiveness *in vivo*, due to the inability of ultraviolet and visible light to penetrate tissue. However, the high degree of user control of these materials has been investigated *in vitro* using microfluidics [214].

Photoresponsive moieties are often implemented in gels as cross-links, or at rare intervals throughout the backbone of hydrogel systems (usually NIPAAm or PEG-based gels). Because of the limited monomer content required to instill a sensitivity to light, photoresponsive gels can be synthesized to exhibit many responses, even responses to multiple wavelengths of light [213, 215]. Photoresponsive pendant groups fall into three major categories: isomerization, degradation and dimerization. Isomerization groups undergo either cis/trans shifts or cyclization reactions. The primary group utilized for isomerization is azobenzenes. When diacrylate azobenzenes are included as cross-links, light in the 400–500 nm range can actuate a cis/trains shift the azo group. This isomerization translates into a shift in hydrophobicity and causes significant changes in the mechanical properties [216]. Cyclization reactions often center around spirobenzoyran, where a cationic quaternary amine reacts with a connected hydroxyl group to form a hexagonal ester that encourages gel collapse due to loss of ionic content and increased pi electron interactions [214]. This reaction is energetically unfavorable, and when the sample is left in the dark, reverts to its original structure. This technology has been utilized in responsive microfluidics, resulting in the generation of real time fluid channels.

Photo-degradable hydrogels almost unanimously utilize nitrobenzene groups, either as a cross-linker or as a mechanism for incorporation of a photo-releasable analyte [213, 215]. Not only do these groups offer an effective means for degradation, but when incorporated as a tetra-functional cross-link, they can exhibit responses to multiple wavelengths, one to degrade the network, and another to release the nitrobenzene groups [217]. The photoresponsiveness of nitrobenzene-based gels can be tailored by modification of the nitrobenzene side groups. For example, some labs have developed gels, which respond to light in the near-infrared region, a benefit for many biomedical applications [218].

Dimerizations or other conjugation reactions offer a mechanism for patterning hydrogels in three dimensions. Photodimerization often involves the inclusion of a coumarin molecule as a pendent in the polymer system [219]. Two coumarin molecules come together to form a cyclobutane ring, and due to intense ring strain, degrade back into the original products in the absence of light. This technology has also been utilized to provide a temporary photo-cross linkable system. Cynnamylidene has been utilized to provide a photocrosslinked system that then degrades in response to a lower wavelength of light [220].

### Other environmentally responsive hydrogels

**Figure 6.**
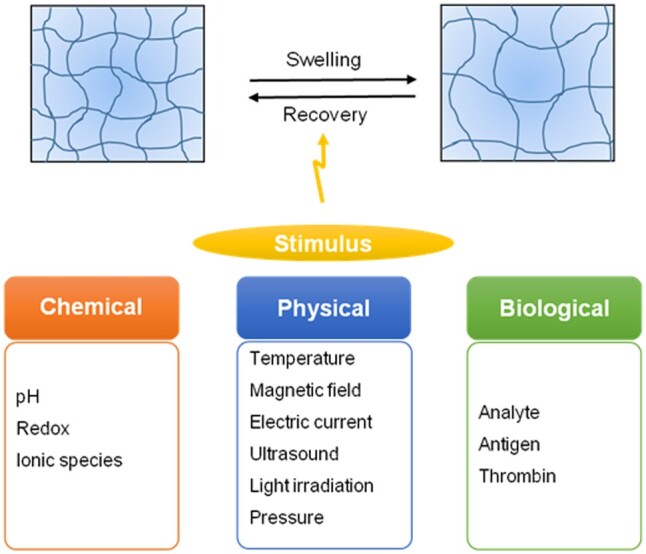
Stimuli-responsive hydrogels. Chemical, physical and biological stimuli have been used to create environmentally responsive hydrogels for various biomedical applications

In addition to the widely used stimuli discussed above, other methods have also been used to create environmentally responsive hydrogels. These can be physical (magnetic field, electric current, ultrasound, light irradiation, pressure or mechanical forces), chemical (ionic species, redox) or biochemical stimuli (antigens, thrombin) (Fig. 6).

#### Electrically responsive hydrogels

Electrically responsive synthetic polymers are often synthesized from polyelectrolytes (polymers, which contain relatively high concentrations of ionizable groups along the backbone chain and are thus, pH-responsive) or electroactive polymers. Natural polymers, such as chondroitin sulfate, agarose, carbomer, xanthan gum and calcium alginate, can be used independently or in conjunction with synthetic polymers, such as acrylate and methacrylate derivatives, to synthesize electrically responsive materials [221]. These hydrogels collapse, swell, bend or erode in response to an applied electric field [195, 196]. In addition to common release due to synereses, diffusion or erosion, electrically responsive charged drugs can be released via electrophoresis toward an oppositely charged electrode [222]. This unique degree of controlled release can also be achieved using non-ionic gels. Polyacrylamide hydrogels connected to both an anode and a cathode undergoes collapse in response to small changes in the electric potential applied across the gel. This results from the migration of H^+^ ions toward the cathode when a potential is applied, which causes loss of water at the anode side. At the same time, electrostatic attraction of negatively charged AA groups toward the anode surface creates a uniaxial stress along the gel axis, mostly at the anode side [223].

Response to electric fields provides precise control via the manipulation of the magnitude of the current, electric impulse duration and interval between pulses. However, the need for a controlled voltage source is a limitation. Drug-delivery systems based on electro-sensitive hydrogels in physiological conditions are rare since most electro-sensitive hydrogels work best in the absence of electrolytes [224]. Electro-responsive hydrogels typically have slow response times, and fatigue over time in response to an increasing number of electrolytes. To overcome this challenge, the size of the gels can decreased by using either micro- or nanoparticles, which exhibit fast response times. Decreasing the size of these gels increases diffusion, which results in enhanced transport in and out of the gel leading to fast response times (a benefit for select biomedical applications).

Composite gels made from conducting polymers and metals/semiconductors combine the unique properties of hydrogels with the electrical and optical properties of the latter. In addition, these composites offer an array of features, such as intrinsic 3D microstructured conducting frameworks, which promote the transport of charged species. An example of this is conductive polyaniline hydrogels made with phytic acid (used as a dopant and gelling agent) that features high electrochemical activity, which can be easily deposited onto surfaces using an ink-jet printer, or simply sprayed [225]. These electrically conductive hydrogels have to potential to facilitate the design of next-generation electronic systems requiring 3D hierarchical nanostructured morphological control, which is envisioned to be useful in several applications, such as medical sensors and implants. As a means to increase the limited number of electro-responsive species, hydrogels incorporating poly(ethyleneimine)-vinyl imidazole were developed by Indermun *et al* [226]. This conductive hydrogel demonstrated on-demand drug release in response to an applied electric field, which ceased upon the removal of the external stimulus.

#### Ultrasound responsive hydrogels

Ultrasound is a promising avenue for drug delivery as it is non-invasive and has a programmable depth of penetration. This allows improved control of drug delivery as the affected area can be targeted by modifying a number of parameters including frequency, power density, duty cycles and time of application. The hydrogel response to ultrasound is related to the generation of thermal energy, perturbation of cell membranes and enhanced permeability of blood capillaries [227]. N-isopropylacrylamide (NIPAM) copolymers were used by Li *et al.* [228] to modify gold nanocages for controlled release using high-intensity focused ultrasound (HIFU). HIFU could rapidly induce a local temperature rise in the focal volume, and thus greatly increased the local release rate, triggered by conventional heating. Huebsch *et al.* [229] demonstrated that alginate hydrogels are capable of reforming in response to damage induced by ultrasound pulses. They hypothesized that ultrasound would disrupt the calcium cross-links, which would then reform due the presence of Ca^2+^*in vivo* thus, facilitating reversible, on-demand release. This strategy revealed to be promising for tumor-targeted delivery of the chemotherapeutic mitoxantrone, since cancer cells are more sensitive to bursts of chemotherapeutics, as opposed to sustained doses.

#### Pressure and mechanically responsive hydrogels

The use of pressure or mechanical stimuli to control drug release from hydrogels has yet to be fully explored. However, since most tissues in the body are subjected to mechanical stimuli, from vessels to muscle and bones, this form of signaling might be advantageous since hydrogels are capable of repeated deformation following compressional loading. The concept that hydrogels may undergo pressure-induced volume phase transition came from thermodynamic calculations based on uncharged hydrogel theory. Hydrogels, which are collapsed at low pressure would expand at higher pressures [223]. The pressure responsive property of hydrogels seems to be a common characteristic of temperature-responsive hydrogels due to an increase in their LCST value with pressure.

Strategic incorporation of mechano-responsive drug depots within hydrogels might offer the opportunity to not only fine-tune gel mechanics, but also to effectively convert mechanical forces exerted on the gel matrix to biochemical signals with desired spatial distributions. For instance, inflamed tissues are routinely exposed to compression, associated with edema and could benefit from inflammation-induced release of anti-inflammatory drugs. Xiao *et al.* [230] developed hyaluronic acid-based hydrogels containing radically cross-linked block copolymer micelles assembled from an amphiphilic block copolymer consisting of hydrophilic poly(acrylic acid) partially modified with 2-hydroxyethyl acrylate, and hydrophobic poly(n-butyl acrylate). These hydrogels demonstrated sustained release of the anti-inflammatory drug dexamethasone over a prolonged period that was accelerated by intermittently applied external compression. Another application of mechano-responsive hydrogels that has been explored is the release of growth factors in response to mechanical signals to guide tissue formation in mechanically stressed environments. In this context, Lee *et al.* [231] created alginate hydrogel matrices, which release growth factors in response to mechanical signals, to promote blood vessel formation.

#### Ion-responsive hydrogels

The responsiveness of hydrogels to ionic strength is a typical property of polymers containing ionizable groups. Changes in ionic strength can result in changes in the volume of the polymer network, polymer solubility, phase transitions of the polymers and fluorescence quenching kinetics of the chromophores bound to electrolytes [227]. For instance, a non-ionic PNIPAAm hydrogel demonstrated a sharp volume phase transition at a critical concentration of sodium chloride in aqueous solution. The phase transition behavior of positively charged poly(diallyl dimethylammonium chloride) hydrogels is sensitive to the concentrations of sodium iodide in solution [232]. Ju *et al.* [233] developed a thermo-responsive hydrogel with ion-recognition properties prepared by free-radical cross-linking copolymerization of NIPAM and benzo-18-crown-6-acrylamide (BCAm) as host receptor. When the crown ether units of the hydrogel captured Ba^2+^ and formed stable BCAm/Ba^2+^ host–guest complexes, the LCST of the hydrogel increased due to the repulsion among charged BCAm/Ba^2+^ complex groups and osmotic pressure within the hydrogel.

#### Redox-responsive hydrogels

Ambient redox potentials in intracellular compartments are regulated by small molecules and proteins (e.g. glutathione (GSH), cysteine and thiredoxin) [234]. Due to a high intracellular concentration of these thiols, in particular GSH, differences in redox potentials are useful resources for intracellular delivery via switchable hydrogels. Nanogels cross-linked with disulfide bonds undergo reduction reactions in the presence of GSH, leading to degradation [235]. For instance, pH and GSH-responsive nanogels based on poly-*N*-isopropylacrylamide (NIPA), *N*-hydroxyethyl acrylamide and tert-butyl 2-acrylamidoethylcarbamate were synthesized by a microemulsion polymerization method using N,N’-cystamine bisacrylamide as a cross-linking agent. This hydrogel formulation demonstrated great potential for tumor-targeted delivery of paclitaxel, where the lower pH and higher intracellular concentration of GSH (i.e. in the tumor) can trigger drug release from the hydrogels [236].

#### Multi-stimuli-responsive hydrogels

Hydrogel targeting, drug delivery and release can be further tailored by the fabrication of smart materials that possess more than one responsive property thus providing, methods for drug protection, local targeting, controlled release, enhanced drug permeation, enzyme inhibition, imaging and reporting [237–239]. One way to readily synthesize multi-stimuli-responsive materials is by the incorporation of magnetic nanoparticles in hydrogel matrices that already exhibit responsive behavior features, such as pH or temperature.

Hydrogel photonic crystal microparticles (HPCMs) with pH-, temperature-, light- and magnetic-responsive properties were generated through a combination of microfluidic, templating techniques and post-functionalization processing. Temperature- and pH-responsive HPCMs were first prepared by copolymerizing functional monomers, such as NIPAM and MAA. These functionalized HPCMs can respond to the UV/visible light without significantly influencing their temperature and pH response, thus enabling multi-responsive properties in a single particle. The presence of magnetic nanoparticles can also facilitate secondary assembly, which has potential applications in advanced optical devices [240]. Casolaro *et al.* [241] reported the development of pH, temperature and magnetic field sensitive vinyl hydrogels bearing α-amino acid residues (L-phenylalanine, L-valine) and incorporating magnetic nanoparticles of different chemical compositions (CoFe_2_O_4_ and Fe_3_O_4_) for the remote controlled release of doxorubicin. In addition, Yang *et al.* developed ultrasound, pH and GSH-responsive biodegradable nanocapsules for imaging and intravenous drug delivery. The nanocapsules, prepared from poly(methacrylic acid) with biodegradable disulfide cross-linking, were loaded with doxorubicin for cancer therapy and filled with perfluorohexane (PFH) for acoustic droplet vaporization imaging. The uniform 300 nm nanocapsules could easily enter the tumor tissues, not only via the enhanced permeability and retention effect, but also due to the enhanced tumor vessel permeability caused by the ultrasound energy. The ultrasound energy also induced the formation of PFH bubbles to create a strong imaging signal, providing echogenic intravenous drug delivery. Within tumor cells, drug release can be triggered by the low pH of lysosomes, as well as by GSH reduction of the skeletal network of the nanocapsules [242]. A multi-responsive hydrogel system co-assembled from phenylalanine derivative gelator and azobenzene derivative has also been constructed to respond to temperature, pH, host–guest interactions and photo irradiation, and was used for controlled cell encapsulation and release in 3D environments. The incorporation of the azobenzene group in the hydrogel resulted in made for UV-responsive properties with additional host–guest interactions due to α-cyclodextrin molecules. Finally, phenylalanine groups provide the potential to tune the self-assembly of hydrogels by adjusting the pH to enhance gelation. This system may further promote the design of advanced multi-stimuli functional scaffolds for the controlled delivery of various therapeutic biological compounds [243].

### Biological tissue interactions with hydrogels

#### Hydrogels in the oral cavity

Oral drug delivery remains widely considered for drug administration, posing several advantages over alternative routes. Oral administration is non-invasive, enabling patient-controlled administration that can increase both the cost-effectiveness and patient compliance for treatment of chronic diseases. Accordingly, hydrogels have been investigated extensively for targeted delivery by controlling swelling characteristics in response to the biological environment and bioadhesive characteristics. The systems discussed address strategies to achieve targeted delivery to specific sites, including the mouth, stomach, small intestine and colon, but have been also been reviewed more detail elsewhere [3, 244].

Hydrogel uses in the oral cavity have been investigated to the addresses treatment of many local diseases of the mouth, including stomatitis, various infections and cancer. A formulation’s residence time in the oral cavity is dependent on the patient’s salivary flow, speech and mastication. Therefore, research has focused on site-specific adhesion of hydrogels to the mucosal surface of the mouth in order to maximize retention time and achieve local treatment to the oral cavity. Several polymers identified as having mucoadhesive properties for use in the oral cavity include, poly(acrylic acid), chitosan, poly(lactic-co-glycolic acid), carbomer and cellulose derivatives [245–247].

As an example of how the hydrogel structure affects its potential medical uses, we discuss briefly the treatment of xerostomia, or dry mouth [248]. Dry mouth is most often a side effect of taking multiple medications, but also recognized as a side effect of many serious medical conditions, such as Parkinson’s disease and Sjogren’s syndrome. Dry mouth increases patient susceptibility to bacterial and fungal infections due to lack of saliva. Furthermore, severe dry mouth, or mucositis, is often associated as a debilitating side effect of chemotherapy or head and neck radiation, and in severe cases, can interrupt cancer treatment. Liquid mucoadhesive hydrogels formulations, such as the polyvinylpyrrolidone-sodium hyaluronate gel (Gelclair ^®^) [249] or the carbomer homopolymer-based MuGard^®^ [250], are classified as a medical devices by the FDA, and prescribed as an oral rinse to form a protective hydrogel layer over injured mucosa to minimize symptoms.

#### Hydrogels in the GI

Oral delivery methods are most commonly administered to target the GI. However, it is also the most complex route for many types of therapeutics due to poor stability in the acidic gastric environment, susceptibility to degradation by digestive enzymes, and difficulties penetrating the rapidly shedding protective mucus layer of the intestinal epithelium, resulting in poor bioavailability for therapeutic efficacy. Hydrogels have been extensively explored to overcome such barriers. Encapsulation of sensitive therapeutics, including small molecules drugs, proteins and peptides, protects the drug payload. Specific swelling properties and/or surface modification of hydrogel carriers can then tailor site-specific delivery or promote interaction with specific cell populations [251, 252].

The small intestine is of particular interest, despite a substantial population of proteolytic enzymes, due the larger absorptive surface area and shortest transit time. pH-responsive hydrogels have been explored for intestinal delivery due to their ability to exploit the pH gradient in the GI tract, remaining complexed in the acidic environment of the stomach to protect the drug, and swell upon transition into the neutral environment of the intestine. For example, Peppas *et al.* pioneered a class of anionic pH-responsive complexation hydrogels for delivery of proteins in the upper small intestine. The predominant network of interest consists of a MAA polymer backbone with grafted PEG tethers. MAA imparts the pH-responsive behavior due to pendant carboxylic acids that ionize below the pKa of 4.8, promoting hydrogen bonding and subsequent strong network complexation. Upon transition to pH above 4.8 (as in the small intestine), deprotonation facilitates hydrogel swelling via electrostatic repulsion and water imbibition to enable protein diffusion into the local environment. PEG tethers both facilitate swelling and impart mucoadhesion to increase residence time of the carrier. The system was first optimized for oral insulin delivery [4, 253], but has also shown promise for delivery of interferon-β [254] and calcitonin [254, 255]. The hydrogels have since been adapted with varying components and explored for delivery of larger molecular weight proteins, such as growth hormone [102], proteins with lower isoelectric points [45] and even delivery of hydrophobic molecules, like the chemotherapeutic doxorubicin, by inclusion of hydrophobic moieties or nanoparticles [101, 256].

Various naturally derived polymers also demonstrate pH-responsiveness and are similarly suitable for oral delivery applications. Natural polymers pose the additional advantage of inherent biocompatibility and physiochemical properties. Chitosan is a naturally derived, linear polysaccharide, which has desirable mucoadhesive properties. Primary amines in the chemical structure generate a positively charged polymer that imparts inherent mucoadhesion and pH-responsiveness such that chitosan-based hydrogels remain collapsed in a neutral pH, as in the mouth, due to presence of free amino groups, and then swell in an acidic environment, such as the stomach, once amino groups are deionized [257–259]. Selective release into the stomach was demonstrated *in vivo* using a semi-IPN composed of chitosan and poly(ethylene oxide) for gastric delivery of model antibiotics amoxicillin and metronidazole [260]. Additionally, chitosan-based hydrogels are degraded by the microflora in the colon, offering a degradation mechanism for localized release of therapeutics, such as calcitonin [261] and the anti-ulcerative colitis drug 5-aminosalicylic acid [262].

Similarly, alginate is another naturally derived polysaccharide, extracted from brown algae. Exposure to calcium ions cross-links the biopolymer to form a hydrogel in an extremely mild gelation strategy. Therefore, alginate is extremely attractive for encapsulation of sensitive therapeutics or cells in order to maintain activity and viability [263]. Alginate exhibits anionic pH-responsive behavior, remaining collapsed at acidic pH and swelling at neutral pH, and has therefore been used for oral administration of small molecule drugs, such as melatonin [100], model proteins (including bovine serum albumin and vaccine protein Helicobacter pylori urease) [264] or oral vaccines [265]. Recently, a complex of the chitosan derivative N, O-carboxymethyl chitosan (NOCC) and alginate, was investigated for oral delivery of proteins drugs. The model protein, bovine serum albumin, was encapsulated into the network in a neutral, aqueous environment [42] to preserve bioactivity, and was retained by the network in acidic pH and released at neutral pH.

#### Hydrogels in the transmucosal area

Numerous other mucosal tissues in the body, including nasal/respiratory, vaginal, rectal and ocular offer alternative routes for drug delivery in an effective and potentially less invasive manner than injection-based administration. These routes offer relatively high bioavailability for either systemic or local administration and avoid the harsh acidic and enzymatic conditions associated with delivery through the GI tract. However, delivery strategies must overcome the rapid mucus clearance associated with pathogen clearance at mucosal surfaces. The advantages, disadvantages and primary applications of each delivery route are further discussed.

##### Hydrogels in the nasal area

The nasal route of administration poses the advantage of a large absorptive surface area and high vasculature within the nasal mucosa, providing an opportunity for delivery of drugs directly into systemic circulation bypassing first-pass metabolism. However, nasal administration poses similar considerations to overcome as discussed within the GI tract, including mucociliary clearance, enzymatic degradation (though proteolytic activity is lower than at GI sites) and low permeability of the epithelium [266]. Strategies to achieve nasal delivery have included bioadhesive gels with high viscosities which promote increased contact time to enable sustained drug release. Formulations containing chitosan have been of particular interest due to its strong mucoadhesive capacity and ability to enhance absorption by opening tight junctions between epithelial cells [239, 240].

Hazan *et al.* [267] developed a thermo-sensitive hydrogel composed of trimethylated chitosan and PEG capable of undergoing a sol–gel transition at physiologically relevant temperature within minutes. *In vivo* studies in diabetic mice indicated gel residence time exceeding the nasal mucus turnover rate, as well as blood glucose control, demonstrating potential for a daily dosage form of insulin via an alternative route [268]. Wu *et al.* [266] also demonstrated application of a thermo-sensitive gel composed of a quaternized chitosan derivative and PEG for nasal delivery of insulin as a model protein therapeutic. Similarly, the formulation undergoes a transition from a sprayable liquid solution to a viscous hydrogel capable of coating the nasal mucosa within minutes at physiological temperature. The same system was adapted for investigation as an adjuvant-free vaccine against the H5N1antigen [269]. The thermo-sensitive gel was able to induce both substantial mucosal immunity and systemic immunity in comparison to adjuvant vaccination in a murine model, as well as increase immune memory.

An alternative approach to thermo-sensitive sprays or drops for nasal administration, mucosal vaccines have been developed composed of nanometer-sized hydrogels (or nanogels) for inhalation. Kiyono *et al.* investigated the efficacy of a cationic cholesteryl group-bearing pullulan (cCHP) nanogel for induction of systemic and mucosal immune protection against respiratory infections [270]. Recently, mice were protected against lethal challenge with *Streptococcuspneumonia* after being vaccinated vial nasal route [271].

##### Hydrogels in the ocular area

Hydrogels have diverse applications for ocular drug delivery for treating diseases in both the anterior and posterior segments of the eye. Polymeric hydrogel-based contact lenses are ideal for drug delivery to the anterior chamber of the eye due to the direct placement on the cornea. Using contact lens-based methods for ophthalmic drug delivery has several advantages over the traditional eye drops, such as increased bioavailability, longer residence time and uniform and controlled-release profile. The use of hydrogels as contact lens-based ophthalmic drug-delivery systems has been extensively reviewed [272–275].

Hydrogel technologies for drug delivery to the back of the eye, known as the ocular posterior segment, have been developed for treating major retinal diseases (e.g. macular edema, age-related macular degeneration, diabetic retinopathy, etc.). Methods for retinal therapy include intravitreally administered hydrogels (implants or injections) and subconjuctivally administered hydrogels. With the least transport barriers, intravitreal implants/injections can effectively deliver drugs to the retina and choroid, but the implantation/injection process is invasive and can lead to complications. In contrast, subconjunctival/transscleral administration of hydrogels is safe, requiring less invasive procedures, but they are relatively less effective in drug delivery.

Hydrogels either, implantable or injectable, can be used to deliver drugs to the retina via intravitreal route for treating retinal diseases, such as choroidal neovascularization, diabetic macular edema, ischemic neovascularization, inflammatory and infectious processes. Both non-degradable and degradable hydrogels have been used as intravitreal implants for drug delivery [276]. Non-degradable sustained release implants are based on ethylene vinyl acetate, polyvinyl alcohol or silicon, to continuously deliver primarily hydrophobic/lipophilic drugs for months to years [277]. Two examples of commercial non-degradable implants are Vitrasert^TM^ (ganciclovir implant) for cytomegalovirus retinitis and Retisert^TM^ (fluocinolone actinide implant) for chronic non-infectious uveitis [276]. Ozurdex (Allergen, Inc.), a biodegradable poly(D,L-lactide-co-glycolide) (PLGA) intravitreal implant for the sustained release of dexamethasone, is an FDA-approved first-line therapy for treatment of macular edema [278].

While intravitreal injection is the most direct method of delivery to the posterior segment, the procedure is associated with numerous complications, such as retinal tears, infection and reduced drug efficacy due to degradation and neutralization. The incorporation of therapeutic molecules into hydrogels for targeted sustained delivery aims to overcome such issues and increase the time between injections [279, 280]. Thermo-responsive hydrogels have shown much promise in intravitreal injection applications since they undergo thermogelation once injected into the vitreous cavity [276, 279, 280]. For example, hydrogels composed of PNIPAAm cross-linked with poly(ethylene glycol) diacrylate (PEGDA) have shown potential as an ocular drug-delivery system that can deliver proteins, such as immunoglobulin G, bevacizumab and ranibiumab [279] without long-term effects on the retina [280]. Another example of intravitreal injection is a dispersion system of drug-loaded PLGA microspheres in a thermogelling PLGA–PEG–PLGA hydrogel that maintains therapeutically relevant vitreal concentrations of ganciclovir for 2 weeks [281].

Due to their biocompatibility, environmentally responsive swelling, and matrix properties similar to natural extracellular matrix, hydrogels are ideal candidates for subconjunctivally implantable delivery systems for posterior segment of eye diseases [276, 282]. A recent study developed a degradable and thermo-responsive hydrogel, composed of NIPAAm and dextran, as a subconjunctival implant for long-term periocular delivery of insulin to treat diabetic retinopathy [283]. Another strategy for subconjunctival implants uses *in situ* gelation as a minimal invasive procedure. Thermosetting gel systems show promise as a safe and convenient sustained method for delivering proteins in the posterior segment of the eye. ReGel^TM^ (BTG International), a commercially available biodegradable and thermo-responsive drug-delivery system containing a triblock copolymer of PLGA and PEG, has been studied for ocular applications. Injecting a mixture of ReGel^TM^ and ovalbumin into the subconjunctival space resulted in the formation of a hydrogel and the sustained release of protein in *in vivo* studies [284]. While subjunctival implants aim for long-term drug release, transscleral methods are typically for short-term delivery. Drug-loaded hydrogels in transscleral iontophoresis devices, which use a weak electric current to enhance transport of charged drugs across percutaneous tissue, are effective for high drug dosages. Hydrogels, serving as drug reservoirs in such devices, reduce tissue irritation and current interruptions [282].

#### Hydrogels in contact with skin

**Figure 7.**
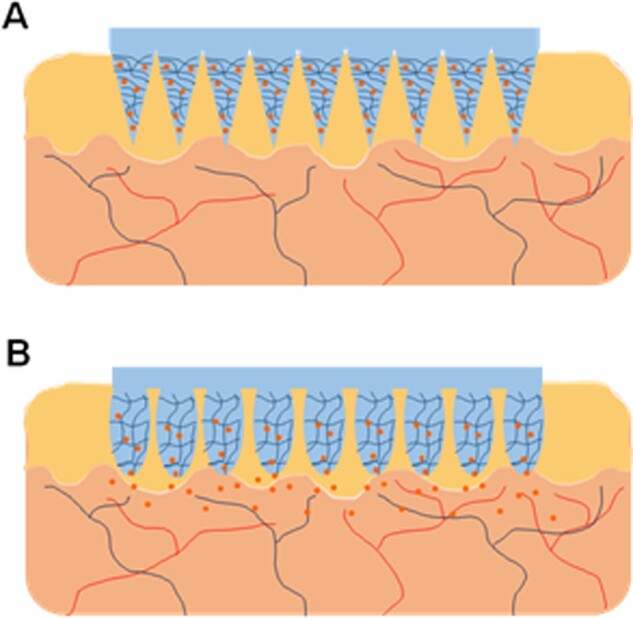
Schematic representation of a swelling hydrogel microneedle array for transdermal drug delivery. Responsive hydrogels can be used to fabricate microneedle array systems for transdermal delivery of drugs (**A**). Upon the application of the stimulus, the swelling of the hydrogel microneedles allows the diffusion of drugs to epidermis and dermis, bypassing the stratum corneum and promoting absorption to blood stream (**B**)

The transdermal route is attractive for drug delivery since it is non-invasive, relatively painless and can be easily self-administered by patients. Moreover, it allows drugs to bypass the first-pass metabolism, decreasing the dose needed for therapeutic effect, consequently decreasing side effects, as well as reducing fluctuations of drug in systemic circulation. The potential benefits for hydrogel transdermal drug delivery include: (i) drugs can be delivered for a long duration at a constant rate, (ii) drug release can be easily interrupted on demand, (iii) released drugs can bypass hepatic first-pass metabolism and (iv) swollen hydrogels can provide a better environment for the skin in comparison to conventional ointments and patches due to higher water content [285]. Traditionally, the delivery of drugs via the skin has been restricted to fairly lipophilic low molecular weight molecules due to the lipophilic nature of skin and skin’s stratum corneum barrier. Several strategies have been explored to bypass this barrier and promote diffusion of drugs into the systemic circulation, such as the use of microneedle arrays (Fig. 7). Microneedles consist of micron-sized projections, typically ranging from 25 to 2000 μm, usually assembled on one side of a supporting base or patch [286]. Donnelly *et al.* [287] described microneedle arrays prepared from the cross-linked polymers (poly(methylvinylether/maleic acid) and PEG), which contain no drug. Instead, they rapidly take up skin interstitial fluid upon skin insertion to form continuous hydrogel conduits from patch-type drug reservoirs to the dermal microcirculation. This system demonstrates that delivery of macromolecules is not limited to what can be loaded into the microneedles themselves. Other strategies can be used to create small microchannels or micropores in the skin for drug penetration, such as thermal, laser and radiofrequency ablation [288].

### Implantable, subcutaneous uses of hydrogels

Subcutaneous exogenous inserted materials may evoke undesirable responses, such as inflammation, carcinogenicity and immunogenicity. Hydrogels also show their applications in subcutaneously implantable therapeutics as they are considered biocompatible materials, due to their high water content, and other promising properties such as: (i) minimal mechanical irritation upon *in vivo* implantation, due to their soft, elastic properties; (ii) prevention of protein adsorption and cell adhesion due to low interfacial tension between water and hydrogels; (iii) broad acceptability for drugs of a wide range of hydrophilicity and molecular sizes; (iv) unique possibility to manipulate the release of incorporated drugs; and (v) biodegradability that avoids the need to remove the implanted hydrogel after the drug has been released [289]. Huang *et al.* [290] successfully used Pd-porphyrins as PEG cross-linkers to generate a polyamide hydrogel with extreme porphyrin density (∼5 mM) to be used as an implantable, oxygen-responsive phosphorescent biomaterial. Liao *et al.* [291] have described an injectable, thermo-responsive hyaluronic acid-*g*-chitosan-*g*-PNIPAAm copolymer for bone tissue engineering. The hydrogel demonstrated a 3D porous structure that allowed the encapsulation of bone marrow-derived mesenchymal stem cells and important features for bone regeneration, such as biocompatibility, bioresorption and ectopic bone formation after injection in mice.

A possible improvement of the present scenario for implantable hydrogels would be the use of an injectable hydrogel formulation, either by using hydrogel particles (microspheres and nanospheres), which could be injected subcutaneously, or polymer solutions that could be injected as a liquid for *in situ* hydrogel polymerization [292, 293]. This would remove the need for surgical implantation of the drug-delivery vehicle and have huge implications regarding cost of treatment and patient acceptability [222]. *In situ* hydrogel polymerization can be induced by several mechanisms, such as solvent exchange [294], photopolymerization [295], ionic cross-linking [296], pH [297] or temperature [298]. For instance, Selvam *et al.* [299] described water-soluble, injectable, biodegradable macromers composed of non-toxic monomers, such as xylitol, maleic acid and PEG, that can cross-link *in situ* by free-radical polymerization using AA as cross-linker, to be used as injectable cell delivery carriers for tissue engineering applications.

### Injectable hydrogels

Hydrogels have shown promise as implantable biomaterials for sustained drug delivery and tissue engineering applications due to their inherent biocompatibility, tissue-like mechanical properties, tunable response and behavior and biodegradability [300–304]. While possessing these desirable qualities, implantable materials have the disadvantage of requiring often invasive and repeated procedures to administer the hydrogel network to the site of interest. Injectable hydrogels, conversely, exhibit all of the benefits of hydrogel networks but are delivered to the site of interest via a non-invasive injection. These networks typically start as an aqueous pre-polymer solution and undergo gelation *in situ* by a variety of mechanisms including temperature, ion concentration, classic organic reaction, enzyme reaction or photoinitiation [298, 304–311]. Injectable systems, in addition to being non-invasive, are also desired because of their ability to form to any cavity with excellent interaction with the surrounding environment and their relatively mild reaction conditions can safely and homogeneously encapsulate precious cargo, such as sensitive therapeutics and cells [312–315]. They are, therefore, excellent candidates for tissue engineering applications. Another subset of injectable gels is so-called shear-thinning hydrogels. These hydrogels are polymerized and fully characterized *ex vivo* but exhibit a shear-thinning behavior, or a significant decrease in viscosity upon the introduction of shear stress, such as that experienced in a needle during injection. This behavior allows solid gels to briefly liquefy during injection and solidify immediately upon cessation of shear [312, 316]. These hydrogels shear thin due to the presence of physical cross-links, such as hydrogen bonding, hydrophobic interactions and electrostatic attraction/repulsion that can be transiently disrupted upon application of a high enough shear force. Self-assembled protein and peptide hydrogels are among the most abundant shear-thinning hydrogels used for tissue engineering applications [312, 317, 318]. Shear-thinning hydrogels, in addition to the benefits associated with other injectable systems, are also able to be fully characterized *ex vivo*. Additionally, these pre-polymerized systems will not be impacted by variations in the physiology of the injection site and will not leach out any potentially inflammatory or cytotoxic species [312].

## Release kinetics

### Models for solute diffusion in hydrogels

Mass transfer in hydrogels is largely governed by Fickian diffusion, since hydrogels are a high percentage of water, the diffusion coefficients of pharmaceutics in gels can be determined as ratios to that of the pharmaceutics in water. The primary difference being the limitations of the free movement of drug by the polymer chains. This limitation results in reptation of the drug through the hydrogel mesh. Models for predicting diffusion coefficients of solutes in hydrogels fall into three categories, free volume, hydrodynamic and obstruction theory.

#### Free volume theory

Free volume theory assumes that the solute can randomly jump through the polymer matrix at a fixed jump distance, *λ*, and calculates the probability that there will be a hole of sufficient size to accommodate the solute at the specified distance. Reinhart and Peppas developed a model utilizing the average mesh size and polymer volume fraction to determine the diffusion of solutes of known size. The resulting relation is defined in Equation 4.1 [319, 320].

4.1 $$\frac{D_{g}}{D_{o}}=\left(1-\frac{r_{s}}{\xi }\right)e\mathrm{xp}\left(-Y\left(\frac{v_{2,s}}{1-v_{2,s}}\right)\right).$$

Where *D*_g_ and *D*_o_ are the rates of diffusion in gel and water, respectively. The radius of the solute is defined as *r*_s_, mesh size is *ξ* and $v_{2,s}$ is the polymer volume fraction. *Y* represents the ratio of the volume needed for a solute step to that of the volume of solute, calculated as *Y* = *γπλr*_s_/*v*_f, w_, where *γ* is a correction factor for space available to more than one solute molecules, *λ* is the average jump length of the solute and *v*_f, w_ is the volume of free water.

#### Hydrodynamic theory

In aqueous environments, solutes are exposed to frictional forces due to the viscosity of water. These forces can be calculated by the Stokes–Einstein equation. In hydrogel systems the viscous layers begin to interact with polymer chains, resulting in increased friction on diffusing solutes. Hydrodynamic theory attempts to calculate these forces to determine the restriction of mass transfer through polymer networks. Cukier derived Equation 4.2 based on the impact of these forces for diffusion through flexible polymer networks, where *k*_c_ is a constant for the polymer/solute system [321].

4.2 $$\frac{D_{g}}{D_{o}}=\exp\left(-k_{c}r_{s}{v_{2,s}}^{.75}\right).$$

#### Obstruction theory

Much like in free volume theories, obstruction theories assume limitations imparted on the free movement off solute molecules by the linear polymer chains. However, it assumes rigid polymer chains in a lattice network to determine the probability of a solute molecule encountering a polymer chain or free site. Obstruction theories have largely been developed for rigid polymer systems [322].

4.3 $$\frac{D_{g}}{D_{o}}=\exp\left(-0.84{\left(v_{2,s}{\left(\frac{r_{s}+r_{f}}{r_{f}}\right)}^{2}\right)}^{1.09}\right).\;$$

### Drug release from hydrogels

While the above models do a good job of predicting the impact of hydrogels on solute diffusion, the freedom of motion of solutes are affected by other factors including partitioning and osmotic gradients. Partitioning occurs due to favorable solute/polymer interactions, largely seen due to attractive forces, charging and the hydrophobic effect. These factors can be altered to improve loading efficiencies and percentages released [101, 323]. Likewise, when hydrogels contain ionic monomers ionic payloads can be similarly trapped due to electrostatic interaction [45, 104]. These situations, combined with potential glassy transitions, require a method to determine if release is Fickian. Ritger and Peppas [61, 324] developed Equation 4.4 for such a method. Where M*_t_*/M_∞_ is the ratio of drug release at time zero and an infinitely large time(*t*), and *k* and *n* are constants. Fitting the fractional solute release vs. time and determining a value for *n* can illuminate whether mass transfer is Fickian. Where *n* is 0.5, 0.45 and 0.43 for Fickian release from slabs, cylinders and sphere, respectively [61].

4.4 $$\frac{M_{t}}{M_{\infty }}=kt^{n}.$$

## Future directions and important problems to address

The previous analysis indicates important aspects of hydrogel architecture, which is related to methods of preparation and actually using in contact with natural and biological fluids, which are related to surface characteristics. Much progress has been made in the last 50 years but there are still important aspects to be addressed.

### Structure and architecture

From a structural point of view the developed equations and mathematical expressions for the 3D behavior of hydrogels must be further developed to answer important foundational questions, such as:

- What is the influence of multifunctional cross-links on the network structure? How does multifunctionality affect mesh size?
- What is the effect of chain ionic charges on the network?
- Can similar equations/theories be developed for ionic hydrogels or hydrogels with strong hydrogen-bonding interactions?
- Non-Gaussian distribution chain structures must be further developed.
- The swelling theory must be re-examined in poor versus thermodynamically good solvents.

### Interactions with biological fluids and surface characteristics

Here, the previous analysis shows that we are far from understanding the hydrogel structure with biological fluids. It is therefore important to continue working on a number of important problems, such as:

- How do surface decorations affect surface tension?
- Is the behavior the same for multicomponent systems and specifically for multicomponent systems with one polymer hydrogel, one liquid (water) and a number if electrolytes plus large molecular weight proteins?
- Re-examination of the behavior of hydrogels in contact with cells.
- Computational analysis of the effect and optimization of the behavior of systems with multiple active groups.

We hope that the advent of advanced computational techniques will aid in these answers.
